# Automating curation using a natural language processing pipeline

**DOI:** 10.1186/gb-2008-9-s2-s10

**Published:** 2008-09-01

**Authors:** Beatrice Alex, Claire Grover, Barry Haddow, Mijail Kabadjov, Ewan Klein, Michael Matthews, Richard Tobin, Xinglong Wang

**Affiliations:** 1 School of Informatics, University of Edinburgh, 10 Crichton Street, Edinburgh, EH8 9AB, UK

## Abstract

**Background:**

The tasks in BioCreative II were designed to approximate some of the laborious work involved in curating biomedical research papers. The approach to these tasks taken by the University of Edinburgh team was to adapt and extend the existing natural language processing (NLP) system that we have developed as part of a commercial curation assistant. Although this paper concentrates on using NLP to assist with curation, the system can be equally employed to extract types of information from the literature that is immediately relevant to biologists in general.

**Results:**

Our system was among the highest performing on the interaction subtasks, and competitive performance on the gene mention task was achieved with minimal development effort. For the gene normalization task, a string matching technique that can be quickly applied to new domains was shown to perform close to average.

**Conclusion:**

The technologies being developed were shown to be readily adapted to the BioCreative II tasks. Although high performance may be obtained on individual tasks such as gene mention recognition and normalization, and document classification, tasks in which a number of components must be combined, such as detection and normalization of interacting protein pairs, are still challenging for NLP systems.

## Background

**Figure 1:**
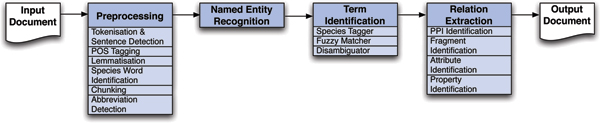
The TXM Pipeline.

Curating biomedical literature into relational databases is a laborious task, in view of the quantity of biomedical research papers that are published on a daily basis. It is widely argued that text mining could simplify and speed up this task [1-3]. In this report we describe how a text mining system developed for a commercial curation project was adapted for the BioCreative II competition. Our submission (team 6) to this competition is based on research carried out as part of the Text Mining (TXM) program, a 3-year project aimed at producing natural language processing (NLP) tools to assist in the curation of biomedical papers. The principal product of this project is an information extraction (IE) pipeline, designed to extract named entities (NEs) and relations relevant to the biomedical domain, and to normalize the NEs to appropriate ontologies (Figure 1). Although the TXM pipeline is designed to assist specialized users, such as curators, it can equally be employed to extract information from the literature that is immediately relevant to biologists in general. For example, it can be used to automatically create large-scale databases or to generate protein-protein interaction networks.

In our BioCreative II submissions, we used the first release of the TXM pipeline, which identifies proteins, normalizes them to a RefSeq derived lexicon, and extracts mentions of protein-protein interactions (PPIs). Since then the pipeline has been extended to identify a wider range of NEs, including proteins, protein complexes, fragments and mutants, modifications, experimental methods, and cell lines. The latest pipeline can predict nested as well as non-nested entities [4]; in other words, it can predict entities that contain, or are contained in, other entities. Furthermore, the PPIs have been enriched [5] with additional information of biological interest, for example whether the PPI is direct or indirect, or what experimental method is used to detect the interaction. In order to demonstrate its adaptability, and to satisfy the needs of the commercial partner, the TXM pipeline was also adapted to the tissue expression domain. In this adaptation, the pipeline was further extended to recognize and normalize an appropriate set of NEs for that domain, such as tissue, protein, mRNA/cDNA, and gene, and to extract and enrich relations that indicate which proteins are expressed in which tissue types.

The TXM pipeline includes both rule-based linguistic preprocessing as well as machine learning (ML)-based IE components, trained on corpora annotated as part of the project. Greater detail regarding the exact implementation of the components is provided in the Materials and methods section (below). The partial reliance on ML is intended to make the pipeline more adaptable, so that it can be easily ported to a different domain if an annotated corpus is available. This adaptability is further enhanced by the use of string distance measures for term normalization, providing a generic method of rapidly comparing the textual form of entities with lexicon entries. Because the pipeline is designed to predict candidate NEs, their normalizations, and PPIs, BioCreative II provided an ideal testing ground to investigate how the pipeline generalizes from its training set. Indeed, one of the largest contributions of BioCreative II is providing training corpora to the research community. These annotated corpora provide common evaluation sets for fair comparison of different text mining algorithms, and provide the means for researchers to develop new ML methods and to encourage researchers in other domains to apply their ML methods to the biological domain.

Our team participated in the following tasks of the competition: gene mention (GM; recognizing gene names); gene normalization (GN; normalizing gene names to EntrezGene identifiers); interaction article subtask (IAS; selecting articles containing curatable PPIs); interaction pair subtask (IPS; extracting curatable PPIs); and interaction sentence subtask (ISS; extracting sentences with evidence for curatable PPIs).

For BioCreative II, and particularly so for the interaction-related tasks, the pipeline could not be used as is, but required certain extensions and modifications. For the IPS subtask, this was because of a fundamental difference between the pipeline's view of a PPI and the PPIs that were to be extracted for BioCreative II. Because the pipeline is intended to be used as a curation assistant, it just attempts to identify the candidate PPI mentions in a document, relying on the human curator to select the curatable PPIs. The definition of a curatable PPI may be somewhat dependent on the curation guidelines in force, but normally refers to PPIs that are experimentally proven in the work described in the paper, as opposed to PPIs that are merely referenced or posited. For the IPS subtask, only curatable PPIs were to be returned, and so additional functionality was implemented on top of the TXM pipeline PPI extraction to remove any extracted but noncuratable PPIs, and to collapse identical PPIs into one.

In the next section we summarize the results of our submissions on each task, and we give some analysis of the performance. This is followed by conclusions drawn from the BioCreative II experience and a description of each of the methods employed. For a comparison of the methods used by all of the participating teams, including our team, see the task overview papers [6-8].

## Results and discussion

### Results

The aim of the GM task was to identify mentions of genes and gene products in sentences extracted from Medline abstracts. As described in the Materials and methods section (below), the submission for the GM task compared two different ML techniques in the three runs, using the same feature set. Runs 1 and 3 employed conditional random fields (CRFs) [9] with different settings of the Gaussian prior, whereas run 2 used a bidirectional maximum entropy Markov model (BMEMM) [10]. (The Gaussian prior is a regularization term applied during learning, to prevent over-fitting. Its value is usually tuned empirically on a held-out set.) The performance of each system, measured by held-out testing on 20% of the training set, and on the test set, is shown in the Table 1.

**Table 1:** Performance in the GM task

Run	Method	Heldout			Test		
		
		Precision	Recall	F_1_	Precision	Recall	F_1_
1	CRF	0.8594	0.8211	0.8398	0.8697	0.8255	0.8470
2	BMEMM	0.8597	0.7982	0.8278	0.8638	0.8041	0.8329
3	CRF	0.8463	0.8297	0.8379	0.8649	0.8248	0.8444

BMEMM, bidirectional maximum entropy Markov model; CRF, conditional random field; GM, gene mention.

The following is an example of the output of the GM system, with the predicted gene mentions highlighted in bold. In this example, the system predicted precisely the same gene mentions as identified by the annotators.

"The **STP1 locus **is located on chromosome IV, close to at least two other genes involved in RNA splicing: **PRP3 **and **SPP41**."

For the GN task, teams were asked to provide a list of EntrezGene identifiers for all of the human genes mentioned in a set of Medline abstracts. We used a string similarity based approximate search algorithm for generating candidate matches for the genes marked up by our GM system. In runs 1 and 2, two variants of an ML-based filter were tested, whereas run 3 used a heuristic filter. The matching and filtering algorithms are described in the Materials and methods section (see below), and Table 2 shows the results obtained on the held-out (20%) training dataset and the test set.

**Table 2:** Performance in the GN task

Run	Method	Heldout			Test		
		
		Precision	Recall	F_1_	Precision	Recall	F_1_
1	ML filter 1	0.681	0.561	0.612	0.767	0.601	0.674
2	ML filter 2	0.674	0.561	0.615	0.767	0.606	0.677
3	Heuristics filter	0.531	0.605	0.566	0.597	0.782	0.677

GN, gene normalization; ML, machine learning.

Submissions were made for three of the four PPI subtasks: the IAS, the IPS, and the ISS. All of these tasks were related to the identification of interactions in articles from PubMed. In the IAS teams, were asked to select abstracts that described curatable interactions, in the IPS teams had to use the full papers to extract pairs of normalized proteins corresponding to the curatable interactions in the paper, and in the ISS, the aim was to identify the sentences in the full texts that described such interactions.

For IAS only one run was submitted, and the performance on the test set is shown in Table 3.

**Table 3:** Performance in the IAS task

AUC	Precision	Recall	F_1_	Accuracy
0.8554	0.7080	0.8609	0.7770	0.7533

AUC, area under the curve; IAS, interaction article subtask.

For IPS, the three submitted runs varied both in the original data format of the article (HTML or PDF), and the algorithm used to generate the UniProt identifier matches (exact or fuzzy). The performances of each configuration, measured using fivefold cross-validation on the training set, and on the test set, are shown in Tables 4 and 5. Note that the scoring algorithm used on the training set is stricter in that it includes all gold (annotated) interactions, whereas scoring on the test set only includes interactions where protein identifiers are drawn from SwissProt.

**Table 4:** Performance in the IPS task, using tenfold cross-validation on the training set

Run	File type	Normalizer	Micro-averaged			Macro-averaged		
			
			Precision	Recall	F_1_	Precision	Recall	F_1_
1	PDF	Exact	0.2680	0.1712	0.2089	0.1945	0.2162	0.1784
2	HTML	Exact	0.2552	0.1692	0.2035	0.1840	0.2100	0.1708
3	PDF	Fuzzy	0.2336	0.1766	0.2011	0.1901	0.2211	0.1757

IPS, interaction pair subtask.

**Table 5:** Performance in the IPS task, on the test set

Run	File type	Normalizer	Micro-averaged			Macro-averaged		
			
			Precision	Recall	F_1_	Precision	Recall	F_1_
1	PDF	Exact	0.2302	0.1283	0.1648	0.2757	0.3011	0.2532
2	HTML	Exact	0.2003	0.1204	0.1504	0.2218	0.2592	0.2066
3	PDF	Fuzzy	0.2131	0.1496	0.1758	0.2392	0.3035	0.2272

IPS, interaction pair subtask.

To see examples of correctly predicted interactions (true positives) and incorrectly predicted interactions (false positives), consider the document with PubMed identifier 10713104. The system correctly predicted an interaction between *LYN_MOUSE *and *HCLS1_MOUSE*, and incorrectly predicted an interaction between *LYN_HUMAN *and *HCLS1_HUMAN*. In the document, there are many sentences in which the pipeline marked an interaction between the two proteins 'Lyn' and 'HS1', for example in the following:

"Here we show that the hemopoietic-specific protein **HS1 **interacted directly with the SH3 domain of **Lyn**, via its proline-rich region."

The UniProt lexicon contains three different possible exact matches for each of the proteins 'Lyn' and 'HS1', with different species, and so the system had to try to determine which particular species the protein mentions referred to. Out of the five species mentioned in the text (*Escherichia coli*, *Homo sapiens*, *Mus musculus*, *Oryctolagus cuniculus*, and *Saccharomyces cerevisiae*), the system chose *M. musculus *(correctly) for some of the interaction mentions and *H. sapiens *(incorrectly) for other interaction mentions.

Finally, for ISS the performance of the one submitted run is shown in Table 6. A sample sentence identified by the system, from PubMed document 14506250, as showing an interaction between *MO4L1_HUMAN *and *RB_HUMAN*, is as follows:

**Table 6:** Performance in the ISS task

Description	Value
Number of evaluated predicted passages	2,497
Number of evaluated unique passages	2,072
Number of evaluated matches to previously selected	147
Number of evaluated unique matches to previously selected	117
Fraction correct (best) from predicted passages	0.0589
Fraction correct (best) from unique passages	0.0565
Mean reciprocal rank of correct passages	0.5525

ISS, interaction sentence subtask.

"We confirmed the association of **MRGX **with **HDAC1 **by immunoprecipitation/Western analysis and determined that **MRGX **complexes had **HDAC **activity."

The comparison between this sentence and the one selected by the curators attained a similarity score of 0.9574 (on a scale from 0 to 1).

## Discussion

The main observation to be made regarding the results for the GM task is that CRF outperforms BMEMM, using the same feature set, and either evaluated on the official test set or cross-validated on the training set. Although the difference in F_1 _is small (1.2 to 1.4 percentage points), it is noted in [11] that differences of this order can be significant on this dataset. The overall performance of the T6 system on recognizing gene names is competitive with the other submitted systems, although several systems performed significantly better. However, our submission involved a straightforward application of existing technology, there are many easily used CRF implementations available, and the feature set could be assembled and optimized rapidly.

The GN system identifies the entity mentions that have been marked up by GM. Therefore, the recall of the GM system sets an upper-bound for the recall of the GN. It is likely that a GM system optimized toward recall would improve performance of GN. In other words, if GM failed to recognize a gene entity, then there was no way that GN could find an identifier for that gene. Our GM system achieved a recall of 83% on a set of held-out GM training data (see Table 1), and therefore we would expect that the maximum recall of the GN system should be close to that number.

We applied an improved JaroWinkler measure to the GN training dataset and achieved a recall of 85% and a precision of 15%. The JaroWinkler measure is described in the Materials and methods section [below]. To maximize recall, we used a threshold confidence of 0 and took the top two matches. We could not test our GM system on the same dataset for a direct comparison, because gene entities were not marked up in the GN data.

The filter was ML based, and the features that we used in the submitted system are described in the the Materials and methods section (below). We also experimented with other features that were not included in our final system. For example, we obtained 'Google counts' for every name in the supplied gene lexicon, and then assigned Google counts to each identifier by summing up the gene names that associate with the identifier. The assumption was that the Google counts might indicate the popularity of the identifiers, and the less popular ones should be filtered out because they probably occurred rarely in the literature. We also tried the nearest 'species word' as a feature, which might help in filtering out the non-human genes. These features, however, did not improve performance of GN and therefore were not integrated into the final system. One reason that the Google count feature was not helpful was that the world-wide web is noisy, and many gene names are also English common words or other types of proper names, and therefore the counts did not accurately reflect the frequency of occurrences of the gene names. Counts obtained from large biomedical corpora, on the other hand, might help, but more experiments are needed to reach conclusions.

For IAS, the primary goal was to improve the results for article selection by extending the traditional bag-of-words model of text categorization to include features based on NLP. Table 7 compares results of a bag-of-words baseline system to the bag-of-NLP system. For the purposes of comparison, the results are presented for the original test set [see Table 3]. They differ slightly from those obtained for the official test set, which is still to be released by BioCreative II. The baseline system only used the 'word' and 'bigram' features but is otherwise identical to the bag-of-NLP system. The results, presented both for fivefold cross-validation on the training set and for the test set, indicate that the NLP-based features can provide small performance gains. Thus, in comprehensive curation systems that include both an article selection component and an NLP-based assisted curation component, there can be benefits from preprocessing all documents with NLP before article selection as a means of improving the article selection phase. The downside is that a bag-of-NLP system is significantly slower than a bag-of-words system (in our case it is two orders of magnitude slower), although much of the processing can be done off-line.

**Table 7:** Overall results

System	Fivefold cross-validation					Test				
	
	AUC	Precision	Recall	F_1_	Accuracy	AUC	Precision	Recall	F_1_	Accuracy
Baseline	0.9757	0.9452	0.9420	0.9436	0.9276	0.8188	0.6898	0.8480	0.7608	0.7333
Bag-of-NLP	0.9777	0.9550	0.9474	0.9512	0.9374	0.8483	0.6994	0.8747	0.7773	0.7493

AUC, area under the curve; NLP, natural language processing.

**Figure 2:**
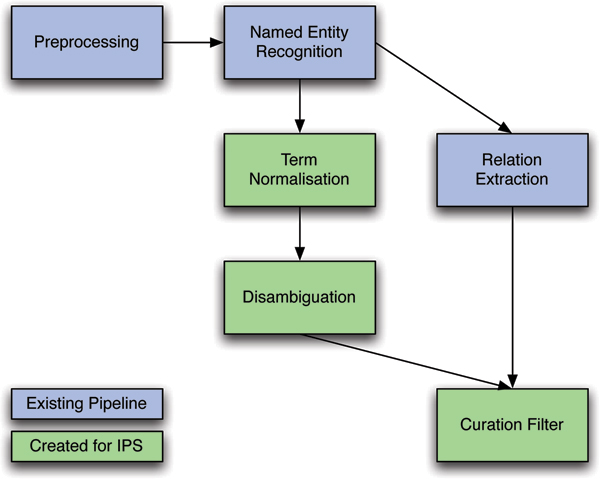
The modification to the TXM Pipeline for the BioCreative IPS Task.

For IPS, several pre-existing TXM pipeline components were used and combined with additional steps to normalize protein names to the UniProt lexicon and to remove noncuratable PPIs. The pipeline is described in detail in the Materials and methods section (see below), but conceptually it can be considered as consisting of the following stages (see Figure 2).

1. Preprocessing: linguistic preprocessing includes tokenization and sentence splitting, lemmatization, chunking, and part-of-speech tagging.

2. Named entity recognition (NER): in this stage all mentions of proteins in the text are identified.

3. In relation extraction (RE), each pair of proteins occurring in the same sentence is examined, and whether the sentence refers to an interaction (PPI) between them is determined.

4. Normalization: in this stage a set of possible UniProt identifiers is generated for each protein mention.

5. The disambiguation stage ranks the set of identifiers produced by the normalization stage, using species information in the text, in order to identify the most likely identifier for each protein.

6. Finally, the curation filter combines the outputs of normalization and RE at a document level to give a list of pairs of UniProt identifiers, representing the PPIs mentioned in the document. The curation filter aims to remove the noncuratable PPIs from this list.

Because the overall system is comprised of several different stages, it would be useful to gain some idea of the performance of each stage to see where improvements could be made.

One way to consider the operation of the pipeline is that the preprocessing, NER, and normalization stages generate a set of possible UniProt identifier pairs, representing curatable interactions, which must then be filtered down by the subsequent three stages. It would therefore be useful to measure the performance of generating curatable PPIs at each stage to determine where improvements can be made. The initial set of UniProt identifier pairs are generated by considering all possible pairs of all possible matches generated for all the proteins found by NER. Consequently, an indication of the recall of each component can be estimated by measuring the number of correct interactions lost at each stage. The normalization requirement in IPS complicates any error analysis, because the gold data, in the form of pairs of UniProt identifiers, are not directly linked to surface forms in the text. However, a certain amount of information about the error sources is available.

In Tables 8 to 11, the results quoted use a version of the IPS training set with all papers with more than 30 interactions removed, which contains 2,039 gold (human curated) interactions. It is expected that similar error patterns would be observed when testing on the test set. Each of the tables shows the number of correctly predicted interactions, together with the total number of predicted interactions, so that the filtering process may be observed as it reduces the number of predictions by removing incorrect interactions, and as a side-effect removes some correct interactions. It is felt that these measures illustrate the filtering process better than the traditional true and false positive and false negative counts, although these counts can easily be derived from the information in Tables 8 to 11.

**Table 8:** Recall of NER and normalization within IPS

File type	Normalization	Correct interactions	% of gold
PDF	Exact	1,204	59.0
HTML	Exact	1,196	58.7
PDF	Fuzzy	1,503	73.7

IPS, interaction pair subtask; NER, named entity recognition.

**Table 9:** Recall of RE within IPS

File type	Normalization	Total interactions	Correct interactions	% of gold	Estimated recall
PDF	Exact	2,052,605	744	36.5	61.7
HTML	Exact	1,916,527	737	36.1	61.6
PDF	Fuzzy	17,583,994	1,002	49.1	66.7

RE, relation extraction; IPS, interaction pair subtask.

**Table 10:** Recall of disambiguator within IPS

File type	Normalization	Total interactions	Correct interactions	% of gold	Estimated recall
PDF	Exact	9,015	495	24.3	66.5
HTML	Exact	8,394	485	23.8	65.8
PDF	Fuzzy	15,383	550	27.0	54.9

IPS, interaction pair subtask.

**Table 11:** Recall of curation filter within IPS

File type	Normalization	Total interactions	Correct interactions	% of gold	Estimated recall
PDF	Exact	953	349	17.1	70.5
HTML	Exact	1,007	345	16.9	71.1
PDF	Fuzzy	1,186	371	18.2	67.5

IPS, interaction pair subtask.

Table 8 shows the percentage of gold interactions for which NER and normalization successfully predicted the identifiers of both participants. Note that the total number of predicted interactions at this point would be equivalent to the count of all pairs of predicted normalizations, and hence is too large to show in the table.

The fuzzy match normalizer generates a much larger number of correct matches than the exact matcher, resulting in increased recall at this stage, although it also generates around ten times more false positives, making the filtering task much harder for the later stages. It is not possible to calculate separate recall figures for the NER and normalizer, because this would require linking each of the gold PPIs to the text, in order to determine whether the NER component had successfully recognized the proteins. Testing of the NER component on the held-out proportion of the TXM corpus gives a recall of about 80% on protein mentions, but the NER task within IPS is different because it only requires the identification of proteins involved in curatable interactions.

The next stage in the pipeline is RE, which takes the output of NER and normalization, examines each pair of proteins, and decides whether the text states that the two proteins interact. Table 9 shows the proportion of gold PPIs that are still extracted after RE, and the total number of proposed PPIs, considering all matches generated by normalization. Furthermore, the estimated recall of RE is given by comparing the number of correct interactions before and after RE. The number of proposed PPIs is large, especially in the fuzzy match configuration, because all possible UniProt matches for each protein have been retained. This means that, for example, if a pair of proteins each has two possible UniProt identifiers, then a total of four different candidate interactions will be generated between them.

In the next stage, the disambiguator chooses the single most likely identifier for each protein mention, using the species information in the text. Table 10 shows the numbers of proposed PPIs, the number of correct and percentage of the gold interactions that are identified, and an estimate of the recall for the disambiguator. It can be seen that the recall of the disambiguator in the fuzzy match configuration is worse; in other words, it throws away more of the correct answers in this configuration. However, it should be remembered that the disambiguator has a much harder task in this case because the number of false positives is much higher, by nearly an order of magnitude. At this point, the difference between the TXM pipeline, which extracts all PPIs, and the task of the BioCreative II challenge of identifying curatable interactions becomes apparent.

The final stage in the pipeline is the curation filter, which is designed to remove noncuratable PPIs from the set of proposed PPIs. Because the curation filter is an ML component trained on the BioCreative II data, fivefold cross-validation was used in the experiments. Its performance is shown in Table 11.

The preceding analysis illustrates one of the issues with the pipeline architecture. Although it provides modularity, which eases development, errors produced by early stages of the pipeline are passed down the pipeline and not corrected by later stages. For example, the disambiguator guesses the species associated with each protein and uses this species to choose the most likely UniProt identifier for the protein from the list proposed by the normalizer. However, if the disambiguator's choices result in a proposed PPI where there is a mis-match between the species of the participating proteins, then that proposed PPI is likely to be discarded by the curation filter. Ideally, the curation filter should be able to feed back to the disambiguator to ask it for alternative identifiers with compatible species. Another example is the interplay between NER and RE. If NER does not predict proteins in a particular sentence, then RE cannot predict a PPI, even if the sentence provides strong linguistic evidence of one. If RE could feedback to NER, then NER would be able to reconsider its decision. However, the possible downside of introducing such feedback between components is that it tends to make the system less modular, and therefore less flexible and maintainable.

In general, the performances of the systems submitted for IPS were low, with no team scoring above 0.3 on macro-averaged F_1_. No equivalent human score, such as an inter-curator agreement, is reported in the literature for comparison. Nevertheless, the level of performance appears to be too low to be usable for unassisted automatic curation. So the question arises, why is the extraction of curatable PPIs so difficult? The above analysis does not single out any component as being especially weak, but suggests that it is the aggregation of errors across the different components that is the problem. The IPS performances should be contrasted to those reported on evaluations that focus on a single task, often making simplifying assumptions, such as only considering human proteins in GN, where performance levels of around 80 to 90% of human performance are often reported.

For ISS the T6 results were quite low, with only 5% of sentences identified agreeing with those selected by the curators. However, it should be noted that the scoring criteria in this subtask are quite strict, in that credit is only given when the system chooses the same evidence sentence as the curator, when it is possible that other sentences from the document would also be appropriate. In order to accurately assess the ISS performance of the submitted systems, it would be necessary to perform an expensive manual examination of all the sentences provided.

## Conclusion

For the PPI subtasks (IPS, ISS, and IAS), the IE pipeline developed for the TXM program proved effective because it addressed related problems (identification of proteins and their interactions) and was trained on similar data to those used in BioCreative II. For IPS the pipeline architecture was easily extended with two extra components (normalization and curation filtering) specific to the requirements of the subtask, showing the flexibility of this architecture. The extension also required a change of emphasis, from a system that aims to assist curators by indicating possible interactions, to a system that attempts to populate a curated database.

Our approach to normalization, based on a string distance measure and ML disambiguation, has the advantage of being more easily adaptable to other types of entities (for example, tissues and cell lines) than the approaches based on manually created matching rules. Given that it is very hard to predict automatically the single correct identifier for a biomedical named entity, it would be interesting to explore the relative merits of approaches that generate a ranked list of candidate identifiers, and also provide the users with fuzzy matching tools to help in searching ontologies more intelligently.

Our submission for IPS involved trying to reconstruct curated information from interactions mentioned explicitly in the text. However, it is not known what proportion of curated data can be obtained this way. In other words, are all or most curatable interactions mentioned explicitly in the text as an interaction between two named proteins? Recent work by Stevenson [12] showed that a significant proportion of facts in the Message Understanding Conference (MUC) evaluations are distributed across several sentences, and similar results appear likely to apply in the biomedical domain. Although the low overall scores in IPS show that NLP techniques are not yet ready to replace manual curation, they may be nevertheless able to aid curators in their work. Alternatively, they may be used to produce large volume, noisy data, which may be of benefit to biologists as evidenced by databases as such as TrEMBL, a computer-annotated database that supplements the manually curated SwissProt database [13].

## Materials and methods

### The TXM pipeline

The Team 6 system for BioCreative II made use of an IE pipeline developed for the TXM project. The TXM pipeline consists of a series of NLP tools, integrated within the LT-XML2 architecture [14]. The development of the pipeline used a corpus of 151 full texts and 749 abstracts selected from PubMed and PubMedCentral as containing experimentally determined protein-protein interactions. The corpus was annotated by trained biologists for proteins and related entities, protein normalizations (to an in-house word list derived from RefSeq), and protein-protein interactions. Around 80% of the documents were used for training and optimizing the pipeline, whereas the other 20% were held back for testing.

The pipeline consists of the following components (see Figure 1).

#### Preprocessing

The preprocessing component comprises tokenization, sentence boundary detection, lemmatization, part-of-speech tagging, species word identification, abbreviation detection, and chunking. The part-of-speech tagging uses the Curran and Clark maximum entropy Markov model tagger [15] trained on MedPost data [16], whereas the other preprocessing stages are all rule-based. The tokenization, sentence boundary detection, species word identification, and chunking components were implemented with the LT-XML2 tools. The Schwartz and Hearst abbreviation extractor [17] was used for abbreviation detection and morpha [18] for lemmatization.

#### Named entity recognition

In the pipeline, NER of proteins is performed using the Curran and Clark classifier [15], augmented with extra features tailored to the biomedical domain. The pipeline NER component was not used in the GM submission, because the pipeline component is trained to detect proteins, and the GM task was concerned with gene products.

#### Term normalization

The term normalization task in the pipeline involves choosing the correct identifier for each protein mention in the text, where the identifiers are drawn from a lexicon based on RefSeq. A set of candidate identifiers is generated using hand-written fuzzy matching rules, from which a single best identifier is chosen using an ML-based species tagger, and a set of heuristics to break ties. The term normalization component of the pipeline was not used directly in BioCreative II because they employ different protein lexicons.

#### Relation extraction

To find the PPI mentions in the text, a maximum entropy relation extractor was trained using shallow linguistic features [19]. The features include context words, parts-of-speech, chunk information, interaction words, and interaction patterns culled from the literature. The relation extractor examines each pair of proteins mentioned in the text, and occurring less than a configurable number of sentences apart, and assigns a confidence value that indicates the degree to which the mention is an interaction. All mentions with a confidence value above a given threshold are considered interactions, whereas those below the threshold are not. Although the relation extractor can theoretically recognize both inter-sentential and intra-sentential relations, because both types of candidate relations are considered, in practice very few inter-sentential relations are correctly recognized. Only around 5% of annotated relations are inter-sentential, and it is likely that using exactly the same techniques as on the intra-sentential relations is not optimal, especially because many of the inter-sentential relations use co-references. The detection of inter-sentential relations is the subject of ongoing research.

The remainder of this section describes how this pipeline was extended and adapted for BioCreative II (see Figure 2), resulting in the best performance per task. Although some time was spent on optimizing parameters and features, the overall infrastructure of the individual TXM pipeline components was applied immediately without significant changes.

### Gene mention

To address the GM task, our team employed two different ML methods using similar feature sets. Runs 1 and 3 used CRFs [9], whereas run 2 used a BMEMM [10]. Both CRF and BMEMM are methods for labeling sequences of words that model conditional probabilities, so that a wide variety of possibly inter-dependent features can be used. The named entity recognition problem is represented as a sequential word tagging problem using the BIO encoding, as in CoNLL (Conference on Computational Natural Language Learning) 2003 [20]. In BMEMM, a log-linear feature-based model represents the conditional probability of each tag, given the word and the preceding and succeeding tags. In CRF, however, the conditional probability of the whole sequence of tags (in one sentence), given the words, is represented using a log-linear model. Both methods have been shown to give state-of-the-art performance in sequential labeling tasks such as chunking, part-of-speech-tagging, and named entity recognition [10,21-23]. The CRF tagger was implemented with CRF++ [24] and the BMEMM tagger was based on Zhang Le's MaxEnt Toolkit [25].

#### Gene mention preprocessing

Before training or tagging the documents with the machine learner, they were passed through the preprocessing stages of the TXM pipeline (as described above).

#### Gene mention features

For the machine learners, the following features were extracted for each word.

1. Word: the word itself is added as a feature, plus the four preceding words and four succeeding words, with their positions marked.

2. Headword: the headwords of noun and verb phrases are determined by the chunker, and, for all words contained in noun phrases, the head noun is added as a feature.

3. Affix: the affix feature includes all character *n*-grams with lengths between two and four (inclusive), and either starting at the first character, or ending at the last character of the word.

4. Gazetteer: the gazetteer features are calculated using an in-house list of protein synonyms derived from RefSeq. To add the gazetteer features to each word in a given sentence, the gazetteer is first used to generate a set of matched terms for the sentence, where each word is only allowed to be in one matched term and earlier starting, longer terms take precedence. The unigram gazetteer feature for each word has value B, I, or O, depending on whether the word is at the beginning, inside, or outside of a gazetteer matched term. The bigram gazetteer feature is also added, and this is the concatenation of the previous and current word's gazetteer feature.

5. Character: for each of the regular expressions listed in Table 12, the character feature indicates whether the word matches the regular expression. These regular expressions were derived from lists published in previous work on biomedical and newswire NER [15,26]. The length of the word is also included as a character feature.

**Table 12:** The (Java) regular expressions used for the character feature in the GM task

Description	Regexp
Capitals, lower case, hyphen then digit	[A-Z]+[a-z]*-[0-9]
Capitals followed by digit	[A-Z]{2,}[0-9]+
Single capital	[A-Z]
Single Greek character	\ p{InGreek}
Letters followed by digits	[A-Za-z]+[0-9]+
Lower case, hyphen then capitals	[a-z]+-[A-Z]+
Single digit	[0-9]
Two digits	[0-9][0-9]
Four digits	[0-9][0-9][0-9][0-9]
Two capitals	[A-Z][A-Z]
Three capitals	[A-Z][A-Z][A-Z]
Four capitals	[A-Z]{4}
Five or more capitals	[A-Z]{5,}
Digit then hyphen	[0-9]+-
All lower case	[a-z]+
All digits	[0-9]+
Nucleotide	[AGCT]{3,}
Capital, lower case then digit	[A-Z][a-z]{2,}[0-9]
Lower case, capitals then any	[a-z][A-Z][A-Z].*
Greek letter name	Match any Greek letter name
Roman digit	[IVXLC]+
Capital, lower, capital and any	[A-Z][a-z][A-Z].*
Contains digit	.*[0-9].*
Contains capital	.*[A-Z].*
Contains hyphen	.*-.*
Contains period	.*\ ..*
Contains punctuation	.*\ p{Punct}.*
All digits	[0-9]+
All capitals	[A-Z]+
Is a personal title	(Mr|Mrs|Miss|Dr|Ms)
Looks like an acronym	([A-Za-z]\.)+

GM, gene mention.

6. Postag: this feature includes the current word's part-of-speech (POS) tag and the POS tags for the two preceding and succeeding words. Also added are the bigram of the current and previous word's POS tag, and the trigram of the current and previous two words' POS tags.

7. Wordshape: the word shape feature consists of the word type feature of [15], and a variant of this feature that only collapses runs of greater than two characters in a word, and bigrams of the word type feature.

8. Abbreviation: the abbreviation feature is applied to all abbreviations whose antecedent is found in the gazetteer.

### Gene normalization

The GN system was developed with genericity in mind. In other words, it can be ported to normalize other biological entities (for example, disease types, experimental methods, and so on) relatively easily, without requiring extensive knowledge of the new domain. The approach that was adopted combined a string similarity measure with ML techniques for disambiguation.

For GN, the system first preprocesses the documents using the preprocessing modules in the TXM pipeline, and then uses the gene mention NER component to mark up gene and gene product entities in the documents. A fuzzy matcher then searches the gene lexicon provided and calculates scores of string similarity between the mentions and the entries in the lexicon using a measure similar to JaroWinkler [27-29].

The Jaro string similarity measure [27,28] is based on the number and order of characters that are common to two strings. Given strings *s *= *a*_1 _... *a*_*k *_and *t *= *b*_1 _... *b*_*l*_, define a character *a*_*i *_in *s *to be shared with *t *if there is a *b*_*j *_in *t *such that *b*_*j *_= *a*_*i *_with *i *- *H *≤ *j *≤ *i *+ *H*, where $\text{H}=\frac{\min(|\text{s}|,|\text{t}|)}{2}$. Let $s^{\prime }={a^{\prime }}_{1}...{a^{\prime }}_{k^{\prime }}$ be the characters in *s *that are shared with *t *(in the same order as they appear in *s*) and let $t^{\prime }={b^{\prime }}_{1}...{b^{\prime }}_{l^{\prime }}$ be analogous. Now define a transposition for *s' *and *t' *to be a position *i *such that ${a^{\prime }}_{i}\neq {b^{\prime }}_{j}$. Let *T*_*s*',*t*' _be half the number of transpositions for *s' *and *t'*. The Jaro similarity metric for *s *and *t *is shown in Equation 1:

(1) $$Jaro(s,t)=\frac{1}{3}\cdot \left(\frac{\left|s^{\prime }\right|}{s}+\frac{\left|t^{\prime }\right|}{t}+\frac{|s^{\prime }|-T_{s^{\prime },t^{\prime }}}{\left|s^{\prime }\right|}\right)$$

A variant of the Jaro measure proposed by Winkler [29] also uses the length *P *of the longest common prefix of *s *and *t*. It rewards strings that have a common prefix. Letting *P' *= *max*(*P*,4), it is defined as shown in Equation 2:

(2) $$JaroWinkler(s,t)=Jaro(s,t)+\frac{P^{\prime }}{10}\cdot (1-Jaro(s,t))$$

For the GN task, a variant of the JaroWinkler measure was employed, as shown in Equation 3, which uses different weighting parameters and takes into account the suffixes of the strings.

(3) $$JaroWinkler^{\prime }(s,t)=Jaro(s,t)+\min(0.99,\frac{P^{\prime }}{10}+\theta )\cdot (1-Jaro(s,t))$$

Here, *θ *= (# *CommonSuffix *- # *DifferentSuffix*)/*lengthOfString*. The idea is to look not only at the common prefixes but also at commonality and difference in string suffixes. A set of equivalent suffix pairs was defined; for example, the Arabic number 1 is defined as equivalent to the Roman number I. The number of common suffixes and the number of different suffixes (1 and 2 or 1 and II would count as different suffixes) is counted, and strings with common suffixes are rewarded whereas those with different ones are penalized.

At the end of the fuzzy matching stage, each mention recognized by NER is associated with the single highest scoring match from the gene lexicon, where the score indicates the string similarity. Note that each match is associated with one or more identifiers (in cases where ambiguity occurs) from the gene lexicon.

The GN system then collects all of the gene identifiers generated by the fuzzy matcher, and pairs each gene identifier with a set of features in order to use an ML-based disambiguator. These identifier-feature set pairs are used as training data to learn a model that predicts the most probable identifier out of a pool of candidates returned by the fuzzy matcher. The feature set consists of both simple features such as the contextual text properties surrounding the gene mentions (for example, their part-of-speech tags and so on), and complex features such as the distance scores between the mentions in text and the matches returned by the fuzzy matcher. It was found that the complex features are particularly helpful in terms of increasing the F_1 _score.

In more detail, all the identifiers in a document found by the fuzzy matcher were collected, then the ones that are correct according to the answer file were used as positive examples and the others were used as negative ones. In summary, each identifier was associated with a set of features as follows.

1. Fuzzy-confidence: confidence scores from the fuzzy matcher. (Only those matches with confidence scores higher than 0.80 were considered.)

2. Synonym-similarity: the averaged confidence score of the similarity between all synonyms linked to the gene identifier and the match.

3. Context-similarity: the similarity between descriptions (synonyms) associated with a gene identifier and all gene entities in the current document recognized by the NER. The similarity is calculated by two measures: dice coefficient and *tf*·*idf*. Dice coefficient is defined as twice the number of common terms in the two sets of tokens to compare, divided by the total number of tokens in both sets: $Dice=\frac{2\times (\#commonTokens)}{\#tokensInSet1+\#tokensInSet2}$. *tf*·*idf *is defined as the product of term frequency (*tf*) and inverse document frequency (*idf*). $tf_{i}=\frac{n_{i}}{\sum_{k}n_{k}}$, where *n*_*i *_is the number of occurrences of the considered term and the denominator is the number of occurrences of all terms. $idf_{i}=\log\frac{\left|D\right|}{\left|\{d:d∍t_{i}\}\right|}$, where |*D*| is the total number of documents and the denominator is the number of documents where the term appears.

4. NER confidence: confidence score generated by the NER tagger.

5. Context: local features, including contextual words (± 10), lemmas (± 4), POS tags (± 2), species words (± 10) and bigrams (± 5). (The numbers in parentheses denote the size of the context window.)

6. Length: length of the gene mention and length of the match.

With the positive and negative examples extracted, determining the correct normalizations becomes a standard ML task. We trained a support vector machine (SVM) classifier, using SVM ^light ^[30], on the examples extracted from the BioCreative II GN training data, and used it as a disambiguator to filter out false-positive identifiers.

### Interaction articles subtask

The IAS was treated as a standard document classification problem [31,32], where abstracts were classified as curatable if they contained curatable protein interaction information and noncuratable otherwise. Document classification techniques typically use a bag-of-words approach, which ignores the word order in the document. This approach was extended by using a 'bag-of-NLP' approach, where, in addition to words, a variety of features derived from the output of the TXM pipeline were added to the bag. The classification was performed with SVM ^light^ using the linear kernel with the default parameters. The documents were ordered based on the output from the SVM classifier.

#### Pipeline processing

Before the documents were passed to the machine learner for training or classification, they were first passed through the the TXM pipeline. In addition, each of the named entities and compound nouns in the document were marked as phrases.

#### Features

The features extracted for each document are described below. Only features that occurred at least twice in the training data were used and each feature was given a binary weight. Each feature was converted to lower case and words found in a custom stop-word list were ignored. For each word a backoff version was also calculated by converting all numbers to a single '#' symbol and removing all punctuation, and a backoff-stemmed version was calculated by first lemmatizing and then performing the same substitutions.

1. Word: the word itself.

2. Word-backoff: the backoff version of the word.

3. Bigram: the bigrams of the backoff feature. The bigrams were not allowed to cross sentence boundaries.

4. Chunk: the concatenation of the backoff-stemmed versions of each word in a chunk up to a maximum of seven words.

5. Phrase: the concatenation of the backoff-stemmed versions of each word in a phrase (one-word phrases were included).

6. Phrase-bigram: the bigrams of the phrase feature. All proteins were converted to the token 'nerprotein'. The bigrams were not allowed to cross sentence boundaries.

7. Chunk-headword-bigram: the bigrams of the backoff-stemmed version of each headword of successive chunks. Chunks containing negative phrases (for instance, does not interact) were indicated by prefixing the bigram with 'neg'.

8. Chunk-headword-trigram: the trigrams of the backoff-stemmed version of each headword of successive chunks. All proteins were converted to the token 'nerprotein'. Chunks containing negative phrases were indicated by prefixing the trigram with 'neg'.

9. Protein: added if the document contained at least one protein.

10. Two-proteins: added if the document contained at least two unique proteins.

11. No-proteins: added if the document did not contain any proteins.

12. Title-proteins: added if the document contained two unique proteins in the title.

### Interaction pairs subtask

The T6 IPS system made use of the TXM IE pipeline to identify mentions of PPIs, together with additional components to normalize proteins to UniProt and to identify the curatable interactions from among the interaction mentions.

#### Data preparation

Two methods of data preparation were used. In runs 1 and 3, the supplied pdftotext converted files were converted to the XML input format required by the pipeline, essentially by just wrapping the text in <text> and <document> elements and removing illegal characters. (These were ASCII control characters inserted by pdftotext, which are not legal in XML. They were all removed except for ASCII 0x0C, which was converted to a double new line.) In run 2, however, the supplied HTML files were used, having been first run through an in-house HTML to XML converter.

#### PPI extraction

The NER and RE stages of the TXM pipeline were used to identify mentions of PPIs.

#### UniProt normalization

Neither the pipeline normalizer nor the GN system could be used directly for normalization: the former because it normalizes to RefSeq, and the latter because it was concerned with genes rather than proteins, and because the IPS required species disambiguation, which was not required for GN. Two approaches were used to assign UniProt identifiers to protein mentions: exact matching (in runs 1 and 2) and fuzzy matching (in run 3). In exact matching, the protein name in the text is compared against each protein synonym in the UniProt lexicon using a case-insensitive match, in order to obtain a list of possible identifiers. If no possible identifiers are found, and the protein name is the long or short form of an abbreviation identified by the abbreviation extractor, then the corresponding (short or long) form is also looked up in the lexicon. In order to filter the list of identifiers, each identifier is weighted according to how often its corresponding species name is mentioned in the text, with species name mentions closer to the protein mention receiving higher weights than those farther away. The identifier with the highest weight is then chosen.

The fuzzy match protein normalizer uses a string distance measure (as described in the GN method description) to find the set of protein names in the lexicon that are closest to the protein mention in the text. These distances are then weighted according to the species word mentions, as for exact matching, and the highest weighted identifier chosen.

#### Curation filter

The curation filter takes as its input the set of UniProt identifier pairs representing the interactions found in the text by the pipeline, with their UniProt normalizations, and outputs the set of normalized, curatable interactions. The filter was implemented with an SVM classifier (using [30] with an RBF kernel), trained on the supplied training data, using the following set of features.

1. Relation count: this feature counts the number of times that the interaction is mentioned in the document.

2. Inter-sentential: this indicates whether the majority of the mentions of the interaction are inter-sentential relations between proteins, or intra-sentential. As noted in the TXM pipeline description, the relation extractor does not perform well on inter-sentential relations, and so very few of these are predicted (only 15 in the training corpus).

3. Relation confidence: each interaction mention found by the pipeline has an associated confidence. The value of this feature is the maximum confidence assigned to an interaction's mentions.

4. Position: this feature specifies the relative position within the document of the first and last mentions of the interaction. In addition, the mean relative position of the interaction mentions is included, for each interaction.

5. Species: the species feature indicates whether the proteins in the proposed interaction have different species.

6. Title: this feature indicates whether the interaction is mentioned in the title.

7. Normalization confidence: when using the fuzzy-matched normalizations, this feature indicates how close a match has been found during normalization of the protein mention.

As recommended in the IPS task instructions, any documents containing more than 30 interactions were excluded from the training set.

### Interaction sentences subtask

To identify the interaction sentences, the T6 system used the same sequence of steps as for IPS. For each interaction pair predicted, the top five corresponding PPI mentions were returned, where PPI mentions were ranked according to the confidence output by the relation extractor. In order to be able to track back the sentences to the original document, the HTML converted data were used (as in IPS run 2), because the HTML to XML converter provided a mapping between the original and converted versions.

## Abbreviations

BMEMM, bidirectional maximum entropy Markov model; CRF, conditional random field; IE, information extraction; GM, gene mention; GN, gene normalization; IAS, interaction article subtask; IPS, interaction pair subtask; ISS, interaction sentence subtask; ML, machine learning; NE, named entity; NER, named entity recognition; NLP, natural language processing; POS, part-of-speech; PPI, protein-protein interaction; RE, relation extraction; SVM, support vector machine.

## Competing interests

This work was funded by ITI Life Sciences, Scotland, whose mission is to explore commercialization of promising technologies in the life sciences.

## Authors' contributions

The TXM pipeline was created by all authors. Within BioCreative II, BH worked on GM and IPS; MM worked on IAS, IPS and ISS; XW worked on GN and IPS; and CG worked on IPS and ISS. All authors contributed to the preparation of the article.

## References

[B1] Yeh AS, Hirschman L, Morgan A (2003). Evaluation of text data mining for database curation: lessons learned from the KDD Challenge Cup.. Bioinformatics.

[B2] Rebholz-Schuhmann D, Kirsch H, Couto F (2005). Facts from text: is text mining ready to deliver?. PLoS Biology.

[B3] Xu H, Krupke D, Blake J, Friedman C (2006). A natural language processing (NLP) tool to assist in the curation of the laboratory mouse tumor biology database.. AMIA Annu Symp Proc.

[B4] Alex B, Haddow B, Grover C (2007). Recognising nested named entities in biomedical text.. Proceedings of BioNLP; Prague, Czech Republic.

[B5] Haddow B, Matthews M (2007). The extraction of enriched protein-protein interactions from biomedical text.. Proceedings of BioNLP, Prague, Czech Republic.

[B6] Smith L, Tanabe LK, Ando R, Kuo CJ, Chung IF, Hsu CN, Lin YS, Klinger R, Friedrich CM, Ganchev K, Torii M, Liu H, Haddow B, Struble CA, Povinelli RJ, Vlachos A, Baumgartner WA, Hunter L, Carpenter B, Tsai RTH, Dai HJ, Liu F, Chen Y, Sun C, Katrenko S, Adriaans P, Blaschke C, Torres R, Neves M, Nakov P, Divoli A, Maña-López M, Mata-Vázquez J, Wilbur WJ (2008). Overview of BioCreative II gene mention recognition.. Genome Biol.

[B7] Morgan AA, Lu Z, Wang X, Cohen AM, Fluck J, Ruch P, Divoli A, Fundel K, Leaman R, Hakenberg J, Sun C, Liu H, Torres R, Krauthammer M, Lau WW, Liu H, Hsu CN, Schuemie M, Cohen KB, Hirschman L (2008). Overview of BioCreative II gene normalization.. Genome Biol.

[B8] Krallinger M, Leitner F, Rodriguez-Penagos C, Valencia A (2008). Overview of the protein-protein interaction annotation extraction task of BioCreative II.. Genome Biol.

[B9] Lafferty J, McCallum A, Pereira F (2001). Conditional random fields: probabilistic models for segmenting and labeling sequence data.. Proceedings of ICML.

[B10] Tsuruoka Y, Tsujii J (2005). Bidirectional Inference with the easiest-first strategy for tagging sequence data.. Proceedings of HLT/EMNLP.

[B11] Wilbur J, Smith L, Tanabe L (2007). BioCreative 2 gene mention task.. Proceedings of the BioCreAtIvE II Workshop; Madrid, Spain.

[B12] Stevenson M (2006). Fact distribution in information extraction.. Lang Resources Eval.

[B13] Bairoch A, Apweiler R (2000). The SWISS-PROT protein sequence database and its supplement TrEMBL.. Nucleic Acids Res.

[B14] Language Technology Group Software. http://www.ltg.ed.ac.uk/software/xml/.

[B15] Curran J, Clark S (2003). Language independent NER using a maximum entropy tagger.. Proceedings of CoNLL03; Edmonton, Canada.

[B16] Smith L, Rindflesch T, Wilbur WJ (2004). MedPost: a part-of-speech tagger for biomedical text.. Bioinformatics.

[B17] Schwartz A, Hearst M (2003). A simple algorithm for identifying abbreviation definitions in biomedical text.. Proceedings of PSB.

[B18] Minnen G, Carroll J, Pearce D (2000). Robust, applied morphological generation.. Proceedings of INLG.

[B19] Nielsen LA (2006). Extracting protein-protein interactions using simple contextual features.. Proceedings of BioNLP; New York, USA.

[B20] Tjong Kim Sang EF, De Meulder F (2003). Introduction to the CoNLL-2003 shared task: language-independent named entity recognition.. Proceedings of CoNLL.

[B21] McCallum A, Li W (2003). Early results for named entity recognition with conditional random fields, feature induction and web-enhanced lexicons.. Proceedings of CoNLL.

[B22] McDonald R, Pereira F (2005). Identifying gene and protein mentions in text using conditional random fields.. BMC Bioinformatics.

[B23] Sha F, Pereira F (2003). Shallow parsing with conditional random fields.. Proceedings of HTL-NAACL.

[B24] http://crfpp.sourceforge.net/.

[B25] Maximum Entropy Modeling Toolkit for Python and C++. http://homepages.inf.ed.ac.uk/s0450736/maxent_toolkit.html.

[B26] Collier N, Takeuchi K (2004). Comparison of character-level and part of speech features for name recognition in biomedical texts.. J Biomed Informatics.

[B27] Jaro MA (1989). Advances in record-linkage methodology as applied to matching the 1985 census of Tampa, Florida.. J Am Stat Assoc.

[B28] Jaro MA (1995). Probabilistic linkage of large public health data files.. Stat Med.

[B29] Winkler WE (1999). The state of record linkage and current research problems.. Tech rep, Statistics of Income Division, Internal Revenue Service Publication R99/04.

[B30] Joachims T (1999). Making large-scale support vector machine learning practical.. Advances in Kernel Methods: Support Vector Machines.

[B31] Donaldson I, Martin J, de Bruijn B, Wolting C, Lay V, Tuekam B, Zhang S, Baskin B, Bader G, Michalickova K, Pawson T, Hogue C (2003). PreBIND and Textomy: mining the biomedical literature for protein-protein interactions using a support vector machine.. BMC Bioinformatics.

[B32] Polavarapu N, Navathe SB, Ramnarayanan R, ul Haque A, Sahay S, Liu Y (2005). Investigation into biomedical literature classification using support vector machines.. Proc IEEE Comput Syst Bioinform Conf.

[B33] Cognia. http://www.cognia.com.

[B34] ITI Life Sciences. http://www.itilifesciences.com.

